# Effects of *Thymus vulgaris* Oil on Sodium Hypochlorite-Induced Damage in Rats

**DOI:** 10.3390/molecules28052164

**Published:** 2023-02-25

**Authors:** Güneş Bolatli, Fatih Taş, Naci Ömer Alayunt

**Affiliations:** 1 Department of Anatomy, Medical Faculty, Siirt University, Siirt 56100, Turkey; 2 Department of Histology and Embryology, Medical Faculty, Siirt University, Siirt 56100, Turkey; 3 Department of Medical Biochemistry, Medical Faculty, Siirt University, Siirt 56100, Turkey

**Keywords:** sodium hypochlorite, *T. vulgaris*, inhalation, TNF-α, TAS, TOS

## Abstract

We aimed to determine the potential damage mechanisms of exposure to widely used sodium hypochlorite (NaOCl) and the effects of *Thymus vulgaris* on this exposure. Rats were divided into six groups: control, *T. vulgaris*, 4% NaOCl, 4% NaOCl + *T. vulgaris*, 15% NaOCl, and 15% NaOCl + *T. vulgaris*. Serum and lung tissue samples were taken after applying NaOCl and *T. vulgaris* by inhalation twice a day for 30 min for four weeks. The samples were examined biochemically (TAS/TOS), histopathologically, and immunohistochemically (TNF-α). In serum TOS values, the mean of 15% NaOCl was significantly higher than in 15% NaOCl + *T. vulgaris*. This was the opposite in terms of serum TAS values. Histopathologically, there was a significant increase in lung injury in 15% NaOCl; significant improvement was observed in 15% NaOCl + *T. vulgaris*. Immunohistochemically, there was a significant increase in TNF-α expression in both 4% NaOCl and 15% NaOCl; significant decreases were observed in both 4% NaOCl + *T. vulgaris* and 15% NaOCl + *T. vulgaris*. The use of sodium hypochlorite, which is harmful to the lungs and is widely used in homes and industries, should be limited. In addition, using *T. vulgaris* essential oil by inhalation may protect against the harmful effects of sodium hypochlorite.

## 1. Introduction

Sodium hypochlorite (NaOCl) is a chemical compound composed of sodium, oxygen, and chlorine. It is used in the food industry, health care, purification of drinking water, endodontic treatment, disinfection, and bleaching [1,2]. It was used at 3–5% concentration in household disinfectants (bleach) [3], 0.5–10% in endodontic treatment [4], and 10–25% in fabric bleaching [5]. Household disinfectants are especially easy to access and widely used. Due to its strong oxidizing properties, NaOCl can cause significant risks from long-term exposure [6]. NaOCl has been proven to cause renal, gastrointestinal, haematological, and pulmonary toxicity [6], but studies demonstrating the adverse effects of NaOCl inhalation are insufficient [5]. Sodium hypochlorite inhalation exposure is common in households and industry due to using as a disinfectant. For this reason, there is a need for studies that reveal the harmful effects of exposure at different doses, taking into account the common usage areas of NaOCl.

It is stated that NaOCl has harmful effects on the pulmonary system [6]. In women with asthma, long-term use of cleansers containing NaOCl has been reported to increase the neutrophil count and cause airway symptoms [7]. NaOCl combines with the water in the mucous membranes of the respiratory tract to form hypochlorous acid. Hypochlorous acid rapidly produces hydrochloric acid and free oxygen radicals [8]. Despite this mechanism, there are mainly case reports in the literature on NaOCl. Information on inhalation exposure is very limited [5]. Inhalation of NaOCl has been reported to cause symptoms such as upper respiratory tract irritation, nausea and vomiting, coughing, irritation of the eyes and nose, dizziness, shortness of breath, and headaches [9]. Long-term use of bleach can cause asthma-like symptoms in some users without a history of allergy [7].

Plant extracts with positive physiological functions are used to eliminate the negative effects of various agents [10,11,12]. It is known that the components in essential oils (carvacrol, carvone, cinnamaldehyde, citral, psimen, eugenol, limonene, menthol, and thymol) do not pose a health risk for the consumer (Everything Added to Food in the US list) [13]. *Thymus vulgaris* (TV) is a perennial herb from the Lamiaceae family without significant side effects, found in central and southern Europe, Africa, and Asia [14]. The most effective components are thymol and carvacrol. Thymol (2-isopropyl-5-methyl phenol) is a monoterpene phenol, also known as “hydroxy cymene” [13]. Carvacrol (C6H3CH3) is a monoterpenoid phenol and has the characteristic odor of thyme. Carvacrol is safe to consume and is used as a natural additive to replace synthetic antioxidative food additives [15].

The aromatic oil of the *T. vulgaris* plant is thyme oil. It is used in traditional medicine to treat dyspepsia, chronic gastritis, diarrhea, and enuresis. It has antispasmodic, anthelmintic, antibacterial, antifungal, antiviral, anti-protozoan, anti-inflammatory, and antioxidant effects [16,17,18]. It also has antiasthmatic, bronchodilator, and expectorant effects on the respiratory system. In this respect, it is widely used to treat respiratory system diseases such as bronchopulmonary disorders [19].

NaOCl causes an increase in free oxygen radicals in tissues [7], and *T. vulgaris* oil has antioxidant, anti-inflammatory, etc. effects. It is known to positively affect the respiratory system [20]. For this reason, we aimed to determine the potential damage mechanisms of exposure to widely used NaOCl and the effects of *T. vulgaris* oil on this exposure by biochemical [total antioxidant status (TAS) and total oxidant status (TOS)], histopathological, and immunohistochemical (TNF-α) methods.

## 2. Results

The analysis certificate information of *T. vulgaris*, which is used as a preservative against sodium hypochlorite application, is shown in Table 1.

### 2.1. Biochemical Findings

TAS/TOS and OSI measurements showed a significant statistical difference between the groups (*p* < 0.05). According to the results of the Tukey test performed to determine which group the difference originated from, the following were found. 

For the TAS measurement, the means of CG, TVG, and 15% N + TVG were statistically significantly higher than that of 15% NG. For the TOS measurement, the mean of 15% NG was statistically significantly higher than all other groups. For the OSI measurement, the mean of 15% NG was statistically significantly higher than all other groups (Table 2).

### 2.2. Histopathological Findings

**Figure 1:**
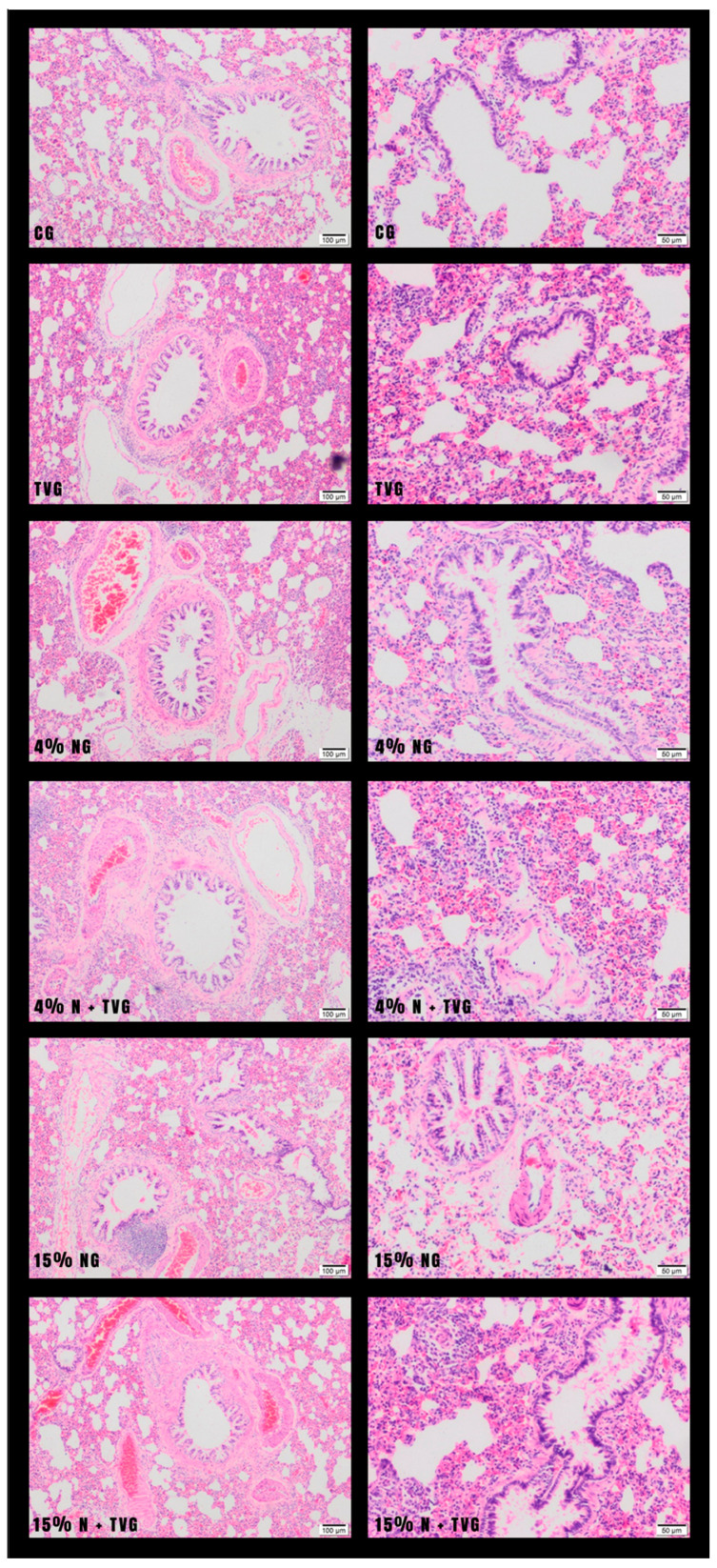
In control and *T. vulgaris* group samples, mostly normal connective tissue formation was observed around the bronchioles. Mild fibrosis was observed in the 4% NaOCl group, and intense fibrosis in the 15% NaOCl group. In addition, inflammatory cells in the alveolar lumens of the lung tissue and inflammatory thickening in the interalveolar septum were detected in the 15% NaOCl group. These findings were not evident in the 4% NaOCl group. It was observed that intense fibrosis, inflammation, and other deterioration in the 15% NaOCl group were significantly reduced with *T. vulgaris* application.

In hematoxylin–eosin (HE) examinations of lung tissue sections, normal lung tissue histology was observed in CG and TVG. While mild fibrosis and inflammation were observed in the lung tissue in 4% NG, significant inflammation and fibrosis were observed in 15% NG. In 4% N + TVG, no significant change was observed in alveolar enlargement and fibrous band formation due to the lesser damage in 4% NG. A significant improvement was observed in 15% N + TVG regarding alveolar enlargement and fibrous band formation compared with 15% NG (Figure 1).

**Figure 2:**
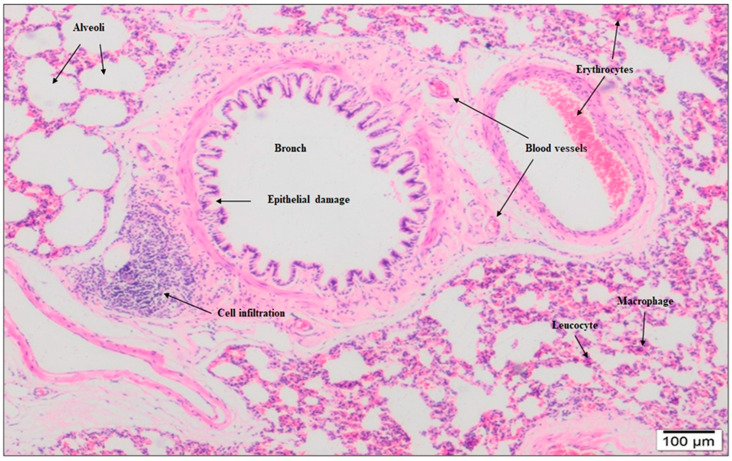
Based on 15% NaOCl application in lung histology, fibrosis was observed in the connective tissue around the vessels, bronchi, and bronchioles. Deteriorations were observed in the terminal and respiratory bronchioles, epithelial and subepithelial connective tissue, and muscle cells below. More peripherally, alveoli were lined with squamous and cuboidal epithelium, and interalveolar septa were found to be of increased thickness. There was local congestion in the veins. Macrophages were occasionally observed in the interalveolar spaces (10× magnification).

In the lung tissue, 15% NG showed localized degeneration of the squamous and cubic cells lining the alveoli, epithelial eruptions in the alveolar spaces, and irregular enlargement due to the merging of the alveolar spaces. In addition, inflammatory thickening and destruction of the interalveolar septum, inflammatory cells in the alveolar lumens, diffuse macrophages, and neutrophils were observed. Intense fibrosis in the lung, significant edema in the bronchiolar epithelium, and the perivascular areas of the vessels accompanying the bronchioles were observed (Figure 2). Although similar histopathological findings were seen in 4% NG, they were not as significant as in 15% NG.

**Figure 3:**
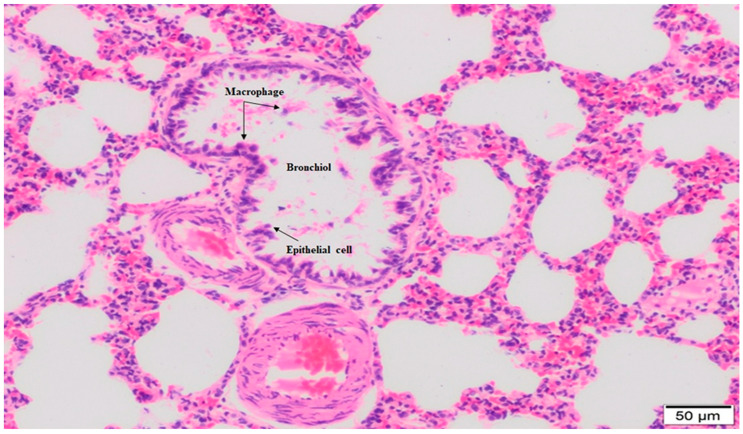
In the lung histology of the 15% NaOCl group, alveolar disruption, infiltrative cells around the vessels, and, in the alveolar area, interalveolar macrophages and neutrophils were observed. Hemorrhage, edema in the interstitial area, and disruption in the interalveolar septum were observed. It was observed that the alveoli were full, the continuity of the epithelium of the respiratory bronchioles was disrupted, and there was an increase in interstitial cells between the alveoli and edema around the vessels (20× magnification).

In the lung tissue of 15% NG examined at large magnification, thickening of the interalveolar septum, increased vessels showing edema and congestion, stasis in the blood vessels, and cell infiltration in the interstitial area were observed. Diffuse interalveolar edema and irregular enlargement were observed in some areas due to the destruction and effacement of interalveolar septa and the merging of alveolar spaces. Type 2 pneumocytes and macrophages spilled into the alveoli were seen. Erythrocytes were observed in many capillary lumens (Figure 3).

**Figure 4:**
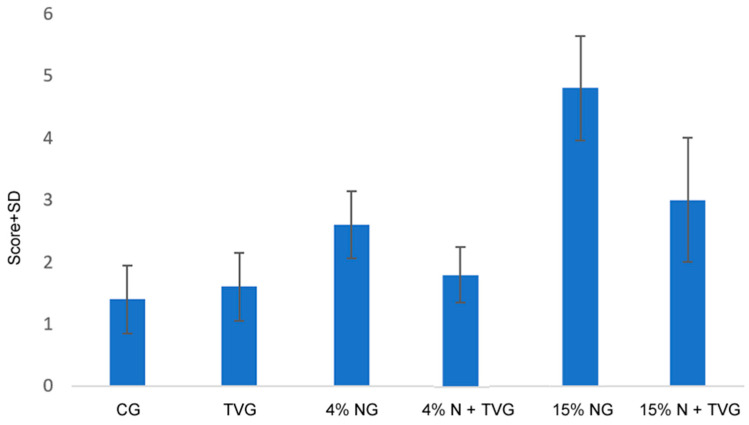
Damage scoring and comparisons of the groups included in the study.

In the damage scoring between the groups, a significant increase was found, especially in 15% NG, and this damage was decreased with *T. vulgaris* application (Figure 4).

This difference between the groups was statistically significant between CG and 15% NG, TVG and 15% NG, 4% NG and 15% NG, and 15% NG and 15% N + TVG (Table 3).

### 2.3. Immunohistochemical Findings

**Figure 5:**
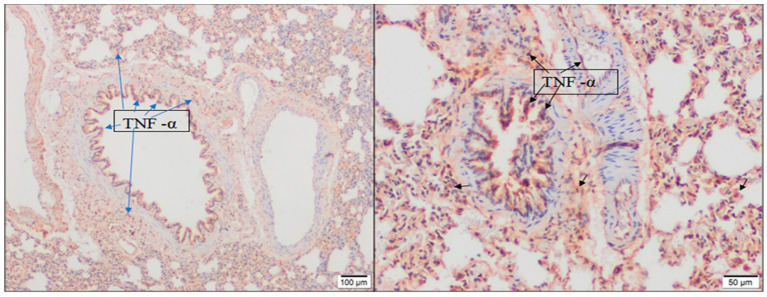
Intense TNF-α expression in alveoli, bronchial and bronchiolar epithelium, inflammatory cells (short black arrow) in lung tissue sections (10× and 20× magnification, respectively) in 15% NaOCl group.

**Figure 6:**
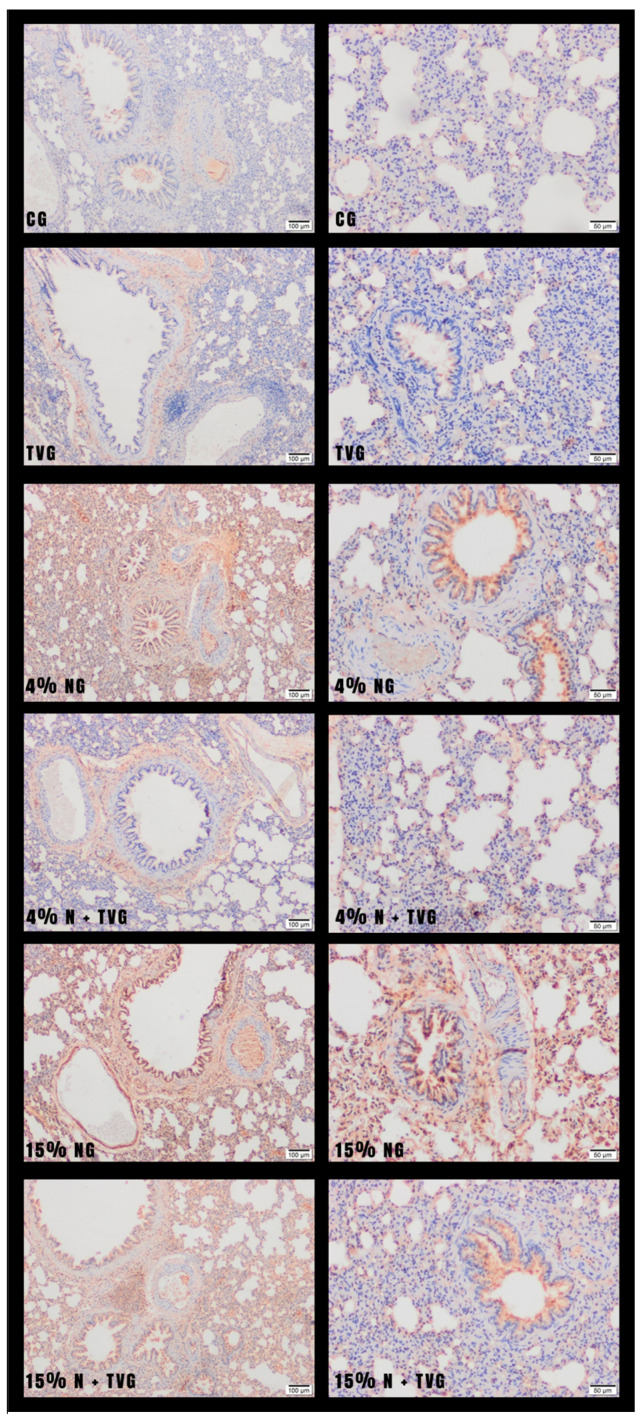
In CG and TVG, mild TNF-α expression was observed in the bronchiolar epithelium. In 4% NG and 15% NG, moderate and intense levels of TNF-α expression were observed in bronchiolar epithelium and macrophages in the alveolar lumen, respectively. 4% N + TVG and 15% N + TVG showed significant decreases in TNF-α expression.

As a result of immunohistochemical staining with TNF-α, mild expression was observed in the lung tissue of the control group and *T. vulgaris* group. In NaOCl-treated groups (4% NG and 15% NG), TNF-α expression was higher in the alveolar, bronchial, and bronchiolar epithelium, as well as in inflammatory cells, especially in alveolar macrophages. TNF-α expression was moderate in 4% NG and intense in 15% NG (Figure 5). TNF-α expression was significantly decreased in 4% N + TVG compared with 4% NG. Similarly, TNF-α expression was significantly decreased in 15% N + TVG compared with 15% NG (Figure 6).

**Figure 7:**
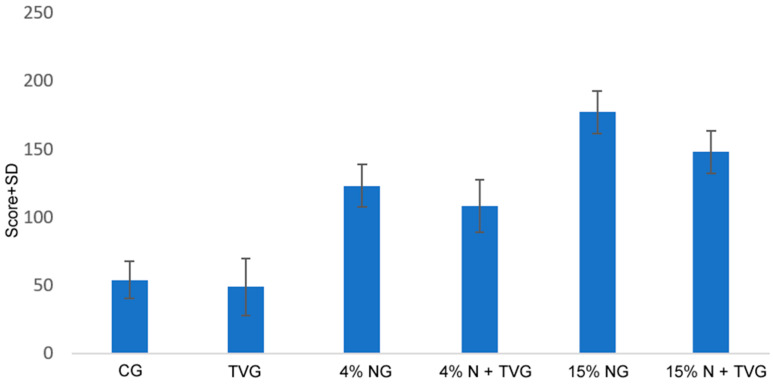
Analysis of the groups in the study with H-score.

In the H-scoring, in which the differences between the groups were evaluated, a significant expression increase was found in 4% NG and 15% NG, and it was observed that the expression levels were significantly decreased in 4% N + TVG and 15% N + TVG (Figure 7).

This difference between the groups was statistically significant between CG and 4% NG, TVG and 4% NG, CG and 15% NG, TVG and 15% NG, 4% NG and 4% N + TVG, and 15% NG and 15% N + TVG (Table 4). 

## 3. Discussion

It is important to investigate the protective effects of natural aromatic oils such as *T. vulgaris*, which are known to have reparative effects on the respiratory system, on the potential damage mechanisms of NaOCl exposure due to inhalation in homes and industries. This study, in which we administered 4% and 15% NaOCl by inhalation, showed that NaOCl negatively affects lung tissue. The protective effect of *T. vulgaris* was examined by looking at rat lung tissues and blood serum levels. Biochemical, histopathological, and immunohistochemical positive effects of *T. vulgaris* application were detected in the 15% NaOCl-treated group. The positive effect of the same application in the group with 4% NaOCl was found to be only immunohistochemically significant.

NaOCl, widespread due to the COVID-19 pandemic, causes side effects in many systems, especially the respiratory system. Long-term exposure, especially to NaOCl inhalation, has been reported to cause severe lower respiratory tract symptoms [21]. NaOCl causes the formation of hydrochloric acid and free oxygen radicals in the mucous membranes of the respiratory tract. This situation causes cytotoxic damage by disrupting cellular proteins [8]. In this study, in accordance with the literature, it was observed that the application of 15% NaOCl statistically increased the TOS level in the serum (*p* < 0.05). Although there was an increase in TOS levels in the 4% NaOCl group, it was not statistically significant. The dose-dependent increase in TOS values in NaOCl exposure suggests that this exposure will cause more damage to the pulmonary system over time.

It is known that exposure to some agents has damaging effects on lung tissue [22,23]. It has been reported that NaOCl inhalation, which can be frequently encountered in routine life, causes mild irritation in the upper respiratory tract at low concentrations; at high concentrations, it causes serious side effects such as pulmonary edema and respiratory distress [5]. In addition, a study investigating the effect of NaOCl inhalation on the trachea found that 4% NaOCl administration caused reversible histopathological effects on the tracheal mucosa [24]. In our study, in accordance with the literature, low-dose NaOCl (4%) inhalation exposure did not cause significant histopathological changes in the lung tissue. Histopathologically, there was a significant increase in the findings of interstitial fibrosis and inflammation in the lung tissues of the group treated with high-dose NaOCl (15%) (*p* < 0.05). In addition, deterioration and irregular enlargement of the alveoli, disruption of the continuity of the bronchi and bronchiole epithelium, hemorrhage, and edema in the interstitial area were observed. Although some histopathological findings were observed in the 4% NaOCl group compared with the control group, it was not as significant as in the 15% NaOCl group. This situation makes us think that NaOCl application in lung tissue can be partially tolerated histopathologically at low doses, but this tolerance disappears when the dose is increased. Therefore, it can be said that the effect on the lung tissue will vary according to the NaOCl application time and concentration.

Although some agents used for various purposes today have beneficial effects, it has been proven by immunohistochemical methods that they cause side effects on some tissues [25]. On the other hand, studies showing the immunohistochemical effects of inhaled NaOCl on lung tissue were not found in the available literature. In our study, the immunohistochemical effects of NaOCl, widely used in households (4%) and industry (15%), on rat lungs were demonstrated by looking at TNF-α expression. A significant increase in expression was detected in both groups (15% group more prominent). This shows that NaOCl causes inflammatory effects at both low and high doses. That the more intense TNF-α expression immunohistochemically in the 15% NaOCl group was accompanied by more prominent histopathological findings (infiltrative cells in the alveolar lumens, inflammatory thickening in the interalveolar septum, widespread macrophages, and neutrophils) supports our opinion.

It is important to take protective measures or develop remedial practices against NaOCl exposure, which has negative effects and is frequently used in daily life. At this point, essential oils with antioxidant properties, such as *T. vulgaris* [22], can protect against the strong oxidizing effect of NaOCl [6]. It has been stated that the main components of *T. vulgaris*, thymol and carvacrol, have beneficial effects on the respiratory system. Thymol is excreted by respiration, and its free form is not found in plasma and urine. This component is known to be used in treating respiratory diseases [26,27]. Carvacrol, a powerful antioxidant, effectively prevents many diseases [28]. Studies have shown that, in addition to its antioxidant properties, carvacrol has anti-inflammatory, antitumor, analgesic, antihepatotoxic and antimicrobial effects [29].

It is stated that *T. vulgaris* can be used by inhalation in respiratory system diseases [27,30]. *Thymus vulgaris* inhalation therapy has increased oxygen saturation in mechanically ventilated patients. This method decreased the concentration of airway secretions and maximum airway pressure. In addition to these, the increase in ciliary activity and gas exchange in the respiratory tract in patients [31] suggests that these applications cause improvement at histopathological appearance.

In our study in which *T. vulgaris* treatment was applied against 4% NaOCl injury, it was observed that *T. vulgaris* had an anti-inflammatory effect on lung tissue. TNF-α expression, which was observed at moderate levels in the 4% NaOCl-treated group, showed a significant decrease with the administration of *T. vulgaris*. However, no significant histopathologic and biochemical differences were observed between these groups. This may be because the harmful effects of NaOCl on lung tissue are more dominant in terms of inflammation. Indeed, in a study investigating the antibacterial effect of *T. vulgaris* essential oil on *Clavibacter michiganensis* subsp. *michiganensis*, it was stated that this essential oil could be used as an antibacterial agent [32]. Another reason may be that the oxidative evaluation was examined in serum. Because NaOCl was administered by inhalation in our study, it can be thought that the first affected tissue will be the lung, and then the blood level will be affected. To understand this, new studies in which TAS and TOS levels will be evaluated in lung tissue can be planned.

In our study, significant improvements were observed in all parameters in the group treated with *T. vulgaris* and 15% NaOCl compared with the group treated with 15% NaOCl. This situation makes us think *T. vulgaris* has reparative effects against tissue damage. The decrease in the histopathological findings in the lung in the 15% NaOCl group with the administration of *T. vulgaris* supports our view. In addition, the decrease in inflammatory (TNF-α expression) and oxidative (TOS) findings in the group given 15% NaOCl, together with the administration of *T. vulgaris*, shows that this essential oil has strong anti-inflammatory and antioxidant effects on lung tissue. The significant increase in TAS values in the serum in 15% NaOCl + *T. vulgaris* group also supports this situation. The healing effects of thymol in *T. vulgaris* on the respiratory system [27] and carvacrol’s antioxidant and anti-inflammatory properties [29] may explain the reason for these positive effects.

## 4. Materials and Methods

### 4.1. Experimental Animals

The entire experimental study was carried out in accordance with the “Guide for the Care and Use of Laboratory Animals,” with the approval of Mersin University animal experiments local ethics committee, numbered 2021/19.

Thirty Wistar albino female rats weighing 200–250 g were used in the experiment. The animals were kept in plastic cages (200 × 350 × 450 mm^3^ in size) in a suitable room prepared for the experiment, providing a constant temperature of 24–25 °C and a twelve-hour (12 h) light-dark cycle. The animals were provided with access to water and standard feed ad libitum. Experiments were started after the animals were kept for one week to adapt to the environment.

### 4.2. Experimental Design

Before the experiment, the experimental animals (5 rats in each cage were randomly selected) were divided into six groups. 

The control group (CG) was exposed to water vapor inhalation at a flow rate of 6 L/min for 30 min twice a day for four weeks.

The *T. vulgaris* group (TVG) was exposed to 0.25 mL *T. vulgaris* inhalation at a flow rate of 6 L/min for 30 min twice a day for four weeks. 

The 4% NaOCl group (4% NG) was exposed to NaOCl (4% concentration) inhalation at a flow rate of 6 L/min for 30 min twice daily for four weeks.

The 4% NaOCl + *T. vulgaris* group (4% N + TVG) was exposed to inhalation of NaOCl (4% concentration) at a flow rate of 6 L/min and 0.25 mL of *T. vulgaris* 6 L/min for 30 min twice daily for four weeks.

The 15% NaOCl group (15% NG) was exposed to NaOCl (15% concentration) inhalation at a flow rate of 6 L/min for 30 min twice daily for four weeks.

The 15% NaOCl + *T. vulgaris* group (15% N + TVG) was exposed to NaOCl (15% concentration) at a flow rate of 6 L/min and 0.25 mL of *T. vulgaris* 6 L/min inhalation for 30 min twice a day for four weeks. 

At the end of four weeks, rats in the experimental groups were administered ketamine hydrochloride at a dose of 80 mg/kg intramuscularly. Following intracardiac blood sampling, rats were sacrificed by cervical dislocation. During the sacrifice, 5 mL of blood was collected from all rats in plain biochemistry tubes with gels, centrifuged at 5000 rpm for 5–10 min, separated into sera, and stored in Eppendorf tubes at −80 °C until the day of analysis. In addition, rat lung tissue samples were removed for histologic examinations after sacrification and fixed in 10% formaldehyde.

### 4.3. NaOCI Preparation and Application

NaOCl (BRTR Chemistry Company, İzmir, Turkey) was prepared at 4% and 15% concentrations and administered twice a day, at 8.30 and 16.30, for 30 min (6 L/min), by inhalation using a nebulizer. Inhalation using a nebulizer has long been used to deliver various substances into the airways and lungs [33].

### 4.4. Preparation and Application of Thymus vulgaris Oil

*Thymus vulgaris*, a perennial medicinal plant, is used for nutrapharmaceutical purposes. This plant, which grows mostly in warm temperate and mountainous regions, occurs in parts of Asia, Europe, and northern Africa [34]. 

Essential oils such as thyme oil are usually used by inhalation, due to their volatile properties. This process is carried out directly or indirectly through the respiratory tract. Due to its irritating and spasmolytic properties on the respiratory tract, the administration of essential oils with a diffuser is a preferred method [35]. Considering similar studies [31], pure *T. vulgaris* oil (Talya Company, Antalya, Turkey), was prepared in 5 drops of 10 mL distilled water and administered by inhalation (TVG, 4% N + TVG and 15% N + TVG groups) using a nebulizer twice a day for 30 min (6 L/min) at 8.30 and 16.30.

Two specially designed cages were used during inhalation procedures [33]. The small cage dimensions were 235 × 325 × 170 mm^3^, and the large cage dimensions were 292 × 440 × 200 mm^3^. Rats were placed in the small cage, the large cage was closed on top of the small cage, and a nebulizer was placed inside the large cage. After the nebulizer was placed in the large cage and subjected for about 5 min, the rats were placed in the large cage together with the small cage. 

### 4.5. Biochemical Applications and Laboratory Analysis

Serum samples were thawed on the day of the experiment, and TAS/TOS levels were measured and OSI (oxidative stress index) parameters were calculated according to the procedure below. Serum TAS (Unit: μmol Trolox Eq/L) level analysis was analyzed colorimetrically in a Siemens Advia 2400 brand automatic analyzer at 660 nm using commercial kits (Rel Assay Diagnostics brand, TEST KIT Catalog no. RL0017 LOT.: RL024) suitable for rats. Serum TOS (Unit: μmol H_2_O_2_ Eq/L) level analysis was analyzed colorimetrically in a Siemens Advia 2400 brand automatic analyzer at 530 nm using commercial kits (Rel Assay Diagnostics brand, ASSAY KIT Catalog no. RL0024 LOT: RL026) suitable for rats. The TOS values of the samples were proportioned to the TAS values in percent and the OSI values were calculated [OSI (arbitrary unit, AU) = (TOS, μmol H_2_O_2_ Eq/L)/(TAS, μmol Trolox Eq/L)].

### 4.6. Histopathological Tissue Follow-Up

Lung tissue samples were first fixed in a 10% neutral buffer formaldehyde solution for light microscopic examination. After fixation, tissue samples were placed in cassettes and washed under running water for 2 h. To remove water, tissues were passed through a series of increasing degrees of alcohol (60%, 70%, 80%, 90%, 96%, and 100%). The tissues were then passed through xylol for transparency and then embedded in paraffin. Sections were taken from the paraffin-embedded tissues with a rotary microtome (RM 2135, Leica Instruments, Nussloch, Germany).

### 4.7. Staining and Damage Scoring with Hematoxylin-Eosin

Sections taken from the experimental groups were kept in an oven at 60 °C for 60 min, and then they were removed from xylol for 3 × 5 min and cleared of paraffin. Afterward, laminas were passed through decreasing series of alcohol (100%, 96%, 90%, 70%) and washed in running water for 1 min, then stained in Harris hematoxylin for 2 min and washed in running water for 2 × 2 min. They were dipped in a 1% ammonia-water mixture and washed in running water for 1 min. Laminas were kept in eosin for 2 min, passed through a series of increasing grades of alcohol (70%, 80%, 96%, 100%), taken into xylol for 2 × 1 min, and closed with entellan. Then, the groups were evaluated by examining them under a light microscope.

Blinded scoring was performed to evaluate the pulmonary lesion. The degree of fibrosis in the lung parenchyma was determined. Fibrosis in the lung interstitium was graded as: 0 = “normal lung”; Grade 1 = “minimal fibrous thickening of the alveolar or bronchial walls”; Grades 2 and 3 = “moderate thickening of the lung walls with no obvious damage to the lung”; Grades 4 and 5 = “increasing fibrosis and fibrous band formation with significant damage to the lung structure”; Grades 6 and 7 = “large fibrous areas and severe structural distortion in the lung” and “honeycomb appearance in the lung”; and Grade 8 = “fibrotic obliteration in the total area” [36]. The difference between the findings obtained and the groups were determined by the one-way-ANOVA test, and *p* < 0.05 was considered statistically significant.

### 4.8. Immunohistochemical Staining

Sections of 4 μm thickness were taken from the lung tissue paraffin blocks on laminas. After the sections were kept in an oven at 60 °C for 1 h, they were deparaffinized by taking xylol for 3 × 5 min. Afterward, laminas were passed through decreasing series of alcohols and rehydrated (100%, 96%, 80%, 70%). The sections were rinsed with distilled water twice for 1 min to purify them from alcohol. In order to unmask the antigen, 1/10 diluted CitratBuffer (PH:6) (AP-9003-999, Thermo Fisher Scientific, Waltham, MA, USA) was applied with the PT Module (A80400012 LabVision, București, Romania). In the IHC Stainer, the laminas were attached to the rack slots and the cover plate. Washing was done with PBS for 5 min. The endogenous peroxidase activity of lung tissues left active with 3% hydrogen peroxide (TA-125-HP, Thermo Scientific, Fremont, CA, USA) for 10 min was blocked. It was washed with PBS. Protein was blocked (TA-125-PBQ, Thermo Fisher Scientific, USA) for 10 min. It was incubated with anti-TNF-α antibody (ab269772 Abcam, Cambridge, UK) (Dilue:1/100) for 2 h. Washing was done with PBS for 5 min. It was incubated in Amplifier Quanto (TL-125-QPB, Thermo Fisher Scientific, Waltham, MA, USA) for 20 min and incubated in HRP Polymer Quanto (TL-125-QPH, Thermo Fisher Scientific, MA, USA) for 30 min. Washing was done with PBS at each step. Staining was performed with DAB Chromogen (TA-125-HA, Thermo Scientific, USA) to identify positive cells. Hematoxylin (HHS32, Chemical Co., St. Louis, MO, USA) was applied for 1 min for floor staining. It was washed with distilled water for 2 × 1 min. The laminas were passed through a series of ascending grades of alcohol (70%, 80%, 96%, 100%), taken into xylol for 2 × 1 min, and closed with entellan.

Immunohistochemical staining results were analyzed with the H-score (possible range 0–300), and the staining rate was graded semiquantitatively. H SCORE = ∑Pi(I + 1). Here, ‘I’ represents the staining intensity (0 = no expression, 1 = light, 2 = medium, 3 = intense), and Pi is the percentage of cells stained for each intensity. Then, the total score was calculated with the formula “(1 + staining intensity/3) × staining ratio” [37].

### 4.9. Statistical Analysis

The conformity of the measurements obtained to the normal distribution was examined with the Kolmogorov–Smirnov test. The means of the measurements according to the groups and whether the difference between these averages was significant was examined by one-way analysis of variance. The Tukey test was used to determine the group that made a significant difference. Analyses were performed with SPSS 20.0 software (IBM, Chicago, IL, USA) at the 95% confidence level.

## 5. Limitations

Since the study was conducted on experimental animals, the number of groups and the duration of the study were limited. Regarding the reliability of the results, the study can be made more comprehensive by increasing the number and study time. Secondly, the study used TNF-α immunohistochemically, and TAS and TOS parameters were used biochemically. The study can be enriched using different inflammatory, oxidant, and antioxidant parameters. Thirdly, the best protective dose can be determined by trying different doses of *T. vulgaris* applications against 4% and 15% NaOCl damage.

## 6. Conclusions

In our study, we showed immunohistochemically that NaOCl has inflammatory effects in lung tissue at both low and high doses. At the same time, it was demonstrated by histopathological and biochemical (TAS and TOS) parameters that NaOCl causes lung tissue damage and oxidation at high doses. In response to these effects of NaOCl, we found that *T. vulgaris* has anti-inflammatory, antioxidant, and tissue damage-reducing effects. In this context, the study is valuable in showing the damage to the lung tissue in NaOCl inhalation exposure and reducing this damage by *T. vulgaris*. In light of these data, we believe that it is important to reconsider the concentrations of NaOCl-containing products commonly used in industry and at home and raise clinicians’ awareness about the complications that may occur against this agent.

## Figures and Tables

**Table 1:** Certificate of analysis of *T. vulgaris*.

Physical & Chemical Specifications			
Assay	Result	Specification	Method
Appearance	Conforms	Liquid	Visual
Color	Conforms	Yellow	Visual
Specific Gravity (25 °C)	0.958	0.9–1.05 g/cm^3^	TLTM003
Refractive Index (25 °C)	1.49071	1.48040–1.49592	TLTM005
Composition	Result	Method	
Thymol	65.3	GC-MS	
Carvacrol	3.44	GC-MS	
Cymene	9.83	GC-MS	
cis-sabine hydrate	0.46	GC-MS	
1-Octen-3-ol	0.8	GC-MS	
Terpinen-4-ol	0.42	GC-MS	
Beta-	1.81	GC-MS	
Microbiology Control	Result	Specification	Method
Total Plate Count	<100 cfu/g	NMT100 cfu/g	Ph.Eur.
Total Yeast&Mold	<100 cfu/g	NMT100 cfu/g	Ph.Eur.
E.coli	Negative	Negative	Ph.Eur.
Salmonella	Negative	Negative	Ph.Eur.
Staphylococcus	Negative	Negative	Ph.Eur.
Solubility in water	Insoluble in aqueous solutions		
Storage	Max 25 °C dark in closed containers ad cups		
Shelf Life	When stored accordingly stable for 3 years		

**Table 2:** Statistical analysis of biochemical results between groups.

Dependent Variable			Average Difference	*p*-Value	95% Confidence Interval	
					Lower Limit	Upper Limit
TAS (µmol/L)	CG	TVG	−0.024000	0.789	−0.207	0.159
		4% NG	0.110000	0.227	−0.073	0.293
		4% N + TVG	0.088000	0.331	−0.095	0.271
		15% NG	0.254000 *	0.009	0.071	0.437
		15% N + TVG	0.014000	0.876	−0.169	0.197
	TVG	CG	0.024000	0.789	−0.159	0.207
		4% NG	0.134000	0.144	−0.049	0.317
		4% N + TVG	0.112000	0.219	−0.071	0.295
		15% NG	0.278000 *	0.005	0.095	0.461
		15% N + TVG	0.038000	0.672	−0.145	0.221
	4% NG	CG	−0.110000	0.227	−0.293	0.073
		TVG	−0.134000	0.144	−0.317	0.049
		4% N + TVG	−0.022000	0.806	−0.205	0.161
		15% NG	0.144000	0.118	−0.039	0.327
		15% N + TVG	−0.096000	0.290	−0.279	0.087
	4% N + TVG	CG	−0.088000	0.331	−0.271	0.095
		TVG	−0.112000	0.219	−0.295	0.071
		4% NG	0.022000	0.806	−0.161	0.205
		15% NG	0.166000	0.074	−0.017	0.349
		15% N + TVG	−0.074000	0.412	−0.257	0.109
	15% NG	CG	−0.254000 *	0.009	−0.437	−0.071
		TVG	−0.278000 *	0.005	−0.461	−0.095
		4% NG	−0.144000	0.118	−0.327	0.039
		4% N + TVG	−0.166000	0.074	−0.349	0.017
		15% N + TVG	−0.240000 *	0.012	−0.423	−0.057
	15% N + TVG	CG	−0.014000	0.876	−0.197	0.169
		TVG	−0.038000	0.672	−0.221	0.145
		4% NG	0.096000	0.290	−0.087	0.279
		4% N + TVG	0.074000	0.412	−0.109	0.257
		15% NG	0.240000 *	0.012	0.057	0.423
TOS (µmol/L)	CG	TVG	0.212000	0.723	−1.007	1.431
		4% NG	−0.090000	0.880	−1.309	1.129
		4% N + TVG	−0.028000	0.963	−1.247	1.191
		15% NG	−2.022000 *	0.002	−3.241	−0.803
		15% N + TVG	−0.612000	0.310	−1.831	0.607
	TVG	CG	−0.212000	0.723	−1.431	1.007
		4% NG	−0.302000	0.614	−1.521	0.917
		4% N + TVG	−0.240000	0.688	−1.459	0.979
		15% NG	−2.234000 *	0.001	−3.453	−1.015
		15% N + TVG	−0.824000	0.176	−2.043	0.395
	4% NG	CG	0.090000	0.880	−1.129	1.309
		TVG	0.302000	0.614	−0.917	1.521
		4% N + TVG	0.062000	0.917	−1.157	1.281
		15% NG	−1.932000 *	0.003	−3.151	−0.713
		15% N + TVG	−0.522000	0.386	−1.741	0.697
	4% N + TVG	CG	0.028000	0.963	−1.191	1.247
		TVG	0.240000	0.688	−0.979	1.459
		4% NG	−0.062000	0.917	−1.281	1.157
		15% NG	−1.994000 *	0.002	−3.213	−0.775
		15% N + TVG	−0.584000	0.333	−1.803	0.635
	15% NG	CG	2.022000 *	0.002	0.803	3.241
		TVG	2.234000 *	0.001	1.015	3.453
		4% NG	1.932000 *	0.003	0.713	3.151
		4% N + TVG	1.994000 *	0.002	0.775	3.213
		15% N + TVG	1.410000 *	0.025	0.191	2.629
	15% N + TVG	CG	0.612000	0.310	−0.607	1.831
		TVG	0.824000	0.176	−0.395	2.043
		4% NG	0.522000	0.386	−0.697	1.741
		4% N + TVG	0.584000	0.333	−0.635	1.803
		15% NG	−1.410000 *	0.025	−2.629	−0.191
OSI	CG	TVG	0.200432	0.723	−0.955	1.356
		4% NG	−0.386843	0.496	−1.542	0.769
		4% N + TVG	−0.155687	0.783	−1.311	1.000
		15% NG	−3.000172 *	0.000	−4.156	−1.845
		15% N + TVG	−0.590038	0.302	−1.745	0.565
	TVG	CG	−0.200432	0.723	−1.356	0.955
		4% NG	−0.587276	0.305	−1.743	0.568
		4% N + TVG	−0.356119	0.531	−1.511	0.799
		15% NG	−3.200605 *	0.000	−4.356	−2.045
		15% N + TVG	−0.790470	0.171	−1.946	0.365
	4% NG	CG	0.386843	0.496	−0.769	1.542
		TVG	0.587276	0.305	−0.568	1.743
		4% N + TVG	0.231157	0.683	−0.924	1.387
		15% NG	−2.613329 *	0.000	−3.769	−1.458
		15% N + TVG	−0.203194	0.720	−1.359	0.952
	4% N + TVG	CG	0.155687	0.783	−1.000	1.311
		TVG	0.356119	0.531	−0.799	1.511
		4% NG	−0.231157	0.683	−1.387	0.924
		15% NG	−2.844485 *	0.000	−4.000	−1.689
		15% N + TVG	−0.434351	0.445	−1.590	0.721
	15% NG	CG	3.000172 *	0.000	1.845	4.156
		TVG	3.200605 *	0.000	2.045	4.356
		4% NG	2.613329 *	0.000	1.458	3.769
		4% N + TVG	2.844485 *	0.000	1.689	4.000
		15% N + TVG	2.410134 *	0.000	1.255	3.566
	15% N + TVG	CG	0.590038	0.302	−0.565	1.745
		TVG	0.790470	0.171	−0.365	1.946
		4% NG	0.203194	0.720	−0.952	1.359
		4% N + TVG	0.434351	0.445	−0.721	1.590
		15% NG	−2.410134 *	0.000	−3.566	−1.255

* *p* < 0.05 group *n*:5, total *n*:30.

**Table 3:** Statistical analysis of biochemical results between groups.

Mean Comparison	Difference	q	*p*-Value
CG vs. TVG	−0.2000	0.6547	>0.05
CG vs. 4% NG	−1.2000	3.9280	>0.05
CG vs. 4% N + TVG	−0.4000	1.3090	>0.05
CG vs. 15% NG	−3.4000	11.1290	<0.001
CG vs. 15% N + TVG	−1.6000	5.2370	<0.05
TVG vs. 4% NG	−1.0000	3.2730	>0.05
TVG vs. 4% N + TVG	−0.2000	0.6547	>0.05
TVG vs. 15% NG	−3.2000	10.4740	<0.001
TVG vs. 15% N + TVG	−1.4000	4.5830	<0.05
4% NG vs. 4% N + TVG	−0.8000	2.6190	>0.05
4% NG vs. 15% NG	−2.2000	7.2010	<0.001
4% NG vs. 15% N + TVG	−0.4000	1.3090	>0.05
15% NG vs. 4% N + TVG	−3.0000	9.8200	<0.001
15% NG vs. 15% N + TVG	−1.8000	5.8920	<0.01
4% N + TVG vs. 15% N + TVG	−1.2000	3.9280	>0.05

**Table 4:** Statistical analysis of H-scoring between groups.

Mean Comparison	Difference	q	*p*-Value
CG vs. TVG	5	0.556	>0.05
CG vs. 4% NG	−102	11.345	<0.001
CG vs. 4% N + TVG	−45	5.005	<0.05
CG vs. 15% NG	−154	17.129	<0.001
CG vs. 15% N + TVG	−99	11.011	<0.001
TVG vs. 4% NG	−107	11.901	<0.001
TVG vs. 4% N + TVG	−50	5.561	<0.01
TVG vs. 15% NG	−159	17.685	<0.001
TVG vs. 15% N + TVG	−104	11.567	<0.001
4% NG vs. 4% N + TVG	57	6.340	<0.01
4% NG vs. 15% NG	−52	5.784	<0.01
4% NG vs. 15% N + TVG	3	0.334	>0.05
15% NG vs. 4% N + TVG	109	12.124	<0.001
15% NG vs. 15% N + TVG	55	6.117	<0.01
4% N + TVG vs. 15% N + TVG	−54	6.006	<0.01

## Data Availability

The datasets used during the current study are available from the corresponding author upon reasonable request.

## Sample Availability

Samples of the compounds are not available from the authors.
